# Childhood neurodevelopmental difficulties and risk of adolescent depression: the role of irritability

**DOI:** 10.1111/jcpp.13053

**Published:** 2019-03-25

**Authors:** Olga Eyre, Rachael A. Hughes, Ajay K. Thapar, Ellen Leibenluft, Argyris Stringaris, George Davey Smith, Evie Stergiakouli, Stephan Collishaw, Anita Thapar

**Affiliations:** ^1^ MRC Centre for Neuropsychiatric Genetics and Genomics Division of Psychological Medicine and Clinical Neurosciences Cardiff University Cardiff UK; ^2^ MRC Integrative Epidemiology Unit Bristol Medical School University of Bristol Bristol UK; ^3^ Population Health Sciences Bristol Medical School University of Bristol Bristol UK; ^4^ Emotion and Development Branch National Institute of Mental Health Bethesda MD USA; ^5^ School of Oral and Dental Sciences University of Bristol Bristol UK

**Keywords:** ALSPAC, neurodevelopmental, irritability, depression, attention‐deficit/hyperactivity disorder, autism

## Abstract

**Background:**

Children with neurodevelopmental disorders are at increased risk of developing depression. Irritability predicts depression in the general population and is common in children with neurodevelopmental disorders. Thus, it is possible that irritability in children with neurodevelopmental disorders contributes to the link with later depression. This study aimed to (a) examine the association between childhood neurodevelopmental difficulties and adolescent depression and (b) test whether irritability explains this association.

**Methods:**

Children with any neurodevelopmental difficulty at the age of 7–9 (*n* = 1,697) and a selected, comparison group without any neurodevelopmental difficulty (*n* = 3,177) were identified from a prospective, UK population‐based cohort, the Avon Longitudinal Study of Parents and Children. Neurodevelopmental difficulties were defined as a score in the bottom 5% of the sample on at least one measure of cognitive ability, communication, autism spectrum symptoms, attention‐deficit/hyperactivity symptoms, reading or motor coordination. The Development and Well‐Being Assessment measured parent‐reported child irritability at the age of 7, parent‐reported adolescent depression at the age of 10 and 13, and self‐reported depression at the age of 15. Depression measures were combined, deriving an outcome of major depressive disorder (MDD) in adolescence. Logistic regression examined the association between childhood neurodevelopmental difficulties and adolescent MDD, controlling for gender. Path analysis estimated the proportion of this association explained by irritability. Analyses were repeated for individual neurodevelopmental problems.

**Results:**

Childhood neurodevelopmental difficulties were associated with adolescent MDD (OR = 2.11, 95% CI = 1.24, 3.60, *p* = .006). Childhood irritability statistically accounted for 42% of this association. On examining each neurodevelopmental difficulty separately, autistic, communication and ADHD problems were each associated with depression, with irritability explaining 29%–51% of these links.

**Conclusions:**

Childhood irritability appears to be a key contributor to the link between childhood neurodevelopmental difficulties and adolescent MDD. High rates of irritability in children with autistic and ADHD difficulties may explain elevated rates of depression in the neurodevelopmental group.

## Introduction

Neurodevelopmental disorders are common (Boyle et al., 2011), typically start in early life, and result in impaired functioning (Howlin, Goode, Hutton, & Rutter, 2004). According to the Diagnostic and Statistical Manual of Mental Disorders, Fifth Edition (DSM‐5), this group includes intellectual disability (ID), communication disorders, autism spectrum disorder (ASD), attention‐deficit/hyperactivity disorder (ADHD), specific learning disorders and motor disorders. There is a scientific rationale for this grouping. First, clinical overlap between these disorders is high (Fombonne, 2003; Ghirardi et al., 2018; Jenson & Steinhausen, 2015; Kadesjo & Gillberg, 2001). These disorders also behave as highly correlated traits. Thus, research that focuses on a single diagnosis (e.g. autism) does not allow for testing the contribution of accompanying neurodevelopmental difficulties. Neurodevelopmental disorders also share common features: they onset early in development, tend to show a steady course and affect males more commonly than females (Bishop & Rutter, 2008; Thapar, Cooper, & Rutter, 2017). There is also strong genetic overlap across different neurodevelopmental problems (Faraone, Ghirardi, Kuja‐Halkola, Lichtenstein, & Larsson, 2017; Ghirardi et al., 2018; Wilcutt, Pennington, & DeFries, 2000). Thus, considering neurodevelopmental disorders together may be useful clinically and for research purposes (Thapar et al., 2017).

Children with neurodevelopmental disorders are at increased risk of later depression (Gadow, Guttmann‐Steinmetz, Rieffe, & DeVincent, 2012; Kim, Szatmari, Bryson, Streiner, & Wilson, 2000; Mammarella et al., 2016; Meinzer et al., 2016). This pattern extends to those with sub‐threshold neurodevelopmental problems (Kanne, Christ, & Reiersen, 2009; Roy, Oldehinkel, Verhulst, Ormel, & Hartman, 2014). Depression in young people with neurodevelopmental disorders is clinically important. For example, in those with ADHD, it is associated with greater impairment in social and academic functioning (Blackman, Ostrander, & Herman, 2005), as well as increased rates for psychiatric hospital admission, suicidality (Biederman et al., 2008) and completed suicide (James, Lai, & Dahl, 2004). Identifying mechanisms that contribute to risk of depression in individuals with neurodevelopmental disorders could inform prevention and treatment strategies; furthermore, recognizing children with neurodevelopmental disorders at highest risk of developing depression could allow early identification and intervention.

One potential mechanism that has attracted growing interest is childhood irritability; this is described as a propensity to react with anger, grouchiness or tantrums disproportionate to the situation (Stringaris & Goodman, 2009a). Irritability is a well‐established feature of oppositional defiant disorder (ODD). Recent studies highlight an irritable dimension of ODD (Stringaris, Cohen, Pine, & Leibenluft, 2009; Rowe, Costello, Angold, Copeland, & Maughan, 2010; Krieger, Polanczyk, et al., 2013; Whelan, Stringaris, Maughan, & Barker, 2013 and Burke et al., 2014). The symptoms that best define this dimension include “often loses temper,” “often touchy or easily annoyed” and “often angry and resentful” (Evans et al., 2017). Irritability, when operationalized in the context of the irritable dimension of ODD or as a separate trait, has been found to predict future emotional disorders and depression (Krieger, Polanczyk, et al., 2013; Rowe et al., 2010; Stringaris & Goodman, 2009a; Vidal‐Ribas, Brotman, Valdivieso, Leibenluft, & Stringaris, 2016; Whelan et al., 2013). Severe irritability is particularly common in children with neurodevelopmental disorders (Shaw, Stringaris, Nigg, & Leibenluft, 2014; Simonoff et al., 2012) and may contribute to later depression in this group. However, research into the role of irritability in the association between neurodevelopmental difficulties and depression is limited.

Cross‐sectional studies find evidence that irritability is associated with depressive symptoms in children with ADHD (Ambrosini, Bennett, & Elia, 2013; Eyre et al., 2017; Seymour et al., 2012). Further, in a clinical ASD sample, the irritable dimension of ODD was associated with emotional problems (Mandy, Roughan, & Skuse, 2014). However, these studies examined the association between irritability and depression prior to the typical age of onset of depressive disorder in adolescence. Their cross‐sectional design precludes any temporal relationship between irritability and depression being established.

Longitudinal population‐based studies have found the association between irritability and depression persists after controlling for ADHD (Burke, Hipwell, & Loeber, 2010; Stringaris et al., 2009), and emotion regulation (a broader construct than irritability) has been shown to mediate the longitudinal association between ADHD and depression (Seymour, Chronis‐Tuscano, Iwamoto, Kurdziel, & MacPherson, 2014). However, longitudinal studies that have specifically examined the role of irritability in the longitudinal association between neurodevelopmental difficulties and depression are lacking.

This study utilized a case‐comparison design with groups selected from a large UK population‐based cohort. Aims were to (a) test for association between childhood neurodevelopmental difficulties and adolescent MDD, and (b) test the hypothesis that childhood irritability contributes to the association between concurrent neurodevelopmental difficulties and later MDD. Finally, as the grouping of neurodevelopmental difficulties includes problems from multiple DSM‐5 diagnostic categories, we assessed whether any particular category was driving the results.

## Methods

### Sample

The Avon Longitudinal Study of Parents and Children (ALSPAC) is a longitudinal population‐based cohort that recruited 14,541 pregnant women resident in Avon, UK, with expected delivery dates between 1 April 1991 and 31 December 1992 (Boyd et al., 2013; Fraser et al., 2013). Of these initial pregnancies, 13,988 children were alive at 1 year.

A total of 1,697 with neurodevelopmental difficulties at the age of 7–9 years and a comparison group of 3,177 with no evidence of any neurodevelopmental difficulties were included in this study (see below). Participants were assessed at multiple time points since recruitment using questionnaire and clinic‐based measures. The study website contains details of all the data that are available through a fully searchable data dictionary (http://www.bris.ac.uk/alspac/researchers/data-access/data-dictionary/).

### Ethical considerations

Ethical approval for the ALSPAC study was obtained from the ALSPAC Ethics and Law Committee and Local Research Ethics Committees. All participants provided written informed consent.

### Measures

#### Childhood neurodevelopmental difficulties

To identify those in the ALSPAC sample with a broad group of neurodevelopmental difficulties, we selected measures that covered each of the six DSM‐5 neurodevelopmental disorder categories (intellectual disability, communication disorders, ASD, ADHD, specific learning disorders and motor disorders). Seven scales from six validated measures, covering symptoms in each of these diagnostic categories, were identified. These measures were all collected between age 7 and 9 years and included both questionnaire and clinic‐based measures. They included the Wechsler Intelligence Scale for Children (WISC‐III) (Wechsler, 1991), the Children's Communication Checklist (CCC) (Bishop, 1998), the Social Communication Disorders Checklist (SCDC) (Skuse, Mandy, & Scourfield, 2005), the Development and Well‐Being Assessment (DAWBA) (Goodman, Ford, Richards, Gatward, & Meltzer, 2000), the Wechsler Objective Reading Dimension (WORD) (Rust, Golombok, Trickey, & Wechsler, 2003) and the Movement Assessment Battery for Children (MABC) (Henderson & Sugden, 1992) (Table 1). If subjects scored in the bottom 5% on at least one measure, they were classified as having “any neurodevelopmental difficulties,” even if data from other measures were missing. A comparison group with no evidence of neurodevelopmental difficulties on any of the seven scales was also identified. Therefore, those in the comparison group were required to have data available on all neurodevelopmental measures. See Figure S1 for a flow chart detailing numbers in the neurodevelopmental difficulties and comparison groups.

**Table 1:** Measures used to identify neurodevelopmental difficulties

Measure	Description of measure used to identify ND difficulties	ND difficulty assessed	Age at completion (*N* with data)	*N* with ND difficulties based on this measure
WISC‐III (Wechsler, 1991)	Test measuring cognitive ability in children Provides full‐scale IQ (range: 45–151)	Intellectual disability	8 years, 6 months (7,037)	321
CCC (Bishop, 1998)	70‐item parent questionnaire assessing children's communication.	Communication disorders	9 years, 7 months	
	(a) Speech and syntax subscales total identifying structural language difficulties (range: 45–70)		(7,544)	(a) 408
	(b) Pragmatic composite score identifying pragmatic language difficulties (range: 96–162)		(7,085)	(b) 355
SCDC (Skuse et al., 2005)	12‐item parent questionnaire assessing child social cognition (range: 0–24).	Autistic spectrum disorder	7 years, 7 months (7,886)	373
DAWBA (Goodman et al., 2000)	Structured diagnostic parent interview based on DSM‐IV diagnoses. ADHD section used to generate child ADHD symptom count (range: 0–18).	ADHD	7 years, 7 months (8,158)	372
WORD (Rust et al., 2003)	Series of tests assessing literacy skills in children. Basic reading subtest used to identify reading impairment (range: 0–50).	Specific learning disorder: impairment in reading	7 years, 6 months (7,606)	401
MABC (Henderson & Sugden, 1992).	Series of tests assessing motor ability. Subtests used: heel to toe walking (balance), placing pegs (manual dexterity) and throwing bean bag into a box (ball skills). Standardized scores available for each subtest (Lingham, Hunt, Golding, Jongmans, & Emond, 2009), allowing a total score to be calculated (range: 0–15).	Motor disorder: developmental coordination disorder	7 years, 6 months (6,682)	305

ADHD, attention‐deficit/hyperactivity disorder; CCC, Children's Communication Checklist; DAWBA, Development and Well‐Being Assessment; MABC, Movement Assessment Battery for Children; ND, neurodevelopmental; SCDC, Social Communication Disorders Checklist; WISC‐III, Wechsler Intelligence Scale for Children; WORD, Wechsler Objective Reading Dimension.

All questionnaire/interview measures completed by parents and all tests completed by children.

As well as deriving one overarching categorical variable identifying those with or without “any neurodevelopmental difficulties,” we generated additional, binary (yes/no), categorical variables for each of the six DSM‐5 neurodevelopmental diagnostic categories (e.g. “ID,” “ADHD” or “ASD” difficulties). These binary categorical variables for each neurodevelopmental difficulty were generated by selecting the bottom 5% for each measure of the ALSPAC sample as a cut point. The specific measures used and the neurodevelopmental difficulty they represent are described in Table 1.

#### Childhood irritability

Irritability was assessed using three symptoms from the ODD section of the age 7 parent Development and Well‐Being Assessment (DAWBA) (Goodman et al., 2000): having temper outbursts, being touchy or easily annoyed and being angry and resentful. These symptoms make up an irritable dimension of ODD (Stringaris & Goodman, 2009b), as previously validated in this sample (Burke et al., 2014). Symptoms occurring over the last 6 months were rated “no more than others” (score 0), “a little more than others” (score 1) and “a lot more than others” (score 2). Our primary measure, total irritable score, ranged from 0 to 6. For descriptive purposes and sensitivity analysis, we also derived a binary measure of any versus no irritability, with the presence of any irritability defined as an irritable score of ≥1. The binary cut‐off was set at ≥1. This represents above average levels of irritability in this sample, and separates those with no irritability from those with any irritability, which is easy to assess in practice.

#### MDD in late childhood/adolescence

The depression section of the DAWBA was completed by parents at the age of 10 and 13 years, and by adolescents at the age of 15 years. DAWBA algorithms were used to generate diagnoses of DSM‐IV MDD in the previous 4 weeks (Goodman, Heiervang, Collishaw, & Goodman, 2011). As depression is episodic and we were interested in whether irritability was associated with any episode of depression across late childhood/adolescence, we chose to define depression as the presence of diagnosis at any of the time points measured. Therefore, information was combined to provide the outcome measure: any diagnosis of MDD between ages 10 and 15.

#### Other study measures

The parent‐reported DAWBA at the age of 7 provided information about depression and anxiety diagnoses. Demographic measures included social class based on mother's occupation, maternal and paternal age at birth of child, and maternal and paternal education (A‐levels or higher vs. those without, that is comparing those with or without education up to at least age 18 years).

### Statistical analyses

Analyses used Stata version 14. Group comparisons used chi‐square analysis for categorical variables and *t* tests for continuously distributed measures.

**Figure 1:**
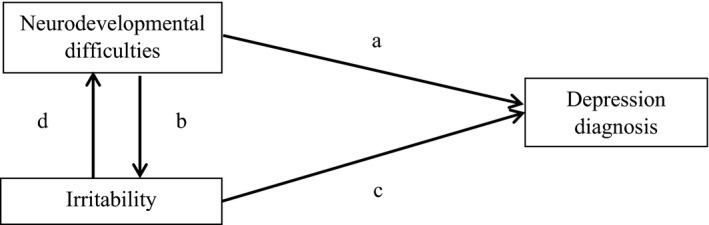
Possible paths between neurodevelopmental difficulties and irritability to depression1. Neurodevelopmental difficulties ⟶ depression (path a)2. Irritability ⟶ depression (path c)3. Neurodevelopmental difficulties ⟶ irritability ⟶ depression (paths b and c)4. Irritability ⟶ neurodevelopmental difficulties ⟶ depression (paths d and a)

Figure 1 shows our proposed path model. Neurodevelopmental difficulties precede the onset of depression and are associated with later depression (Daviss, 2008) (path a, Figure 1). Irritability precedes the onset of depression and is associated with later depression (Stringaris et al., 2009) (path c, Figure 1). Neurodevelopmental difficulties and irritability often co‐occur (Shaw et al., 2014; Simonoff et al., 2012), and the direction of this association is unknown (i.e., path b or path d, Figure 1). However, as neurodevelopmental difficulties start very early in life, and we were interested in the contribution of irritability to the association between neurodevelopmental difficulties and depression, we tested neurodevelopmental difficulties ⟶ depression (path a) and neurodevelopmental difficulties ⟶ irritability ⟶ depression (paths b and c).

Prior to all analyses, those with MDD at the age of 7 years were excluded to ensure we did not include those where the outcome (depression) temporally preceded assessment of the predictor variables.

Logistic regression analysis was used to examine the association between neurodevelopmental difficulties (age 7–9) and MDD (age 10–15) controlling for gender. Gender was included as a covariate as gender is associated with both neurodevelopmental difficulties and depression (Thapar, Collishaw, Pine, & Thapar, 2012; Thapar et al., 2017).

Logistic regression analysis then examined the association between irritability (age 7) and MDD (age 10–15), again controlling for gender.

The association between neurodevelopmental difficulties and irritability at the age of 7–9 was examined by calculating a polychoric correlation coefficient.

To assess the contribution of irritability to the association between childhood neurodevelopmental difficulties and adolescent depression, we used the “khb” command in Stata (Kohler, Karlson, & Holm, 2011) to decompose the path from neurodevelopmental difficulties to depression into direct (path a) and indirect (via irritability—paths b and c) effects, while controlling for gender. This is a general decomposition method that can be used to examine the degree to which a particular variable explains the relationship between an exposure and an outcome, providing information on total, direct and indirect effects. It allows the comparison of coefficients between 2 nested non‐linear probability models (Kohler et al., 2011), which was necessary due to the categorical nature of our variables.

Regression analyses were conducted on both complete case and imputed data sets (see below). As it was not possible for the khb command to be used on multiple imputed data sets in Stata, path analysis was conducted on complete cases only.

Finally, as our neurodevelopmental difficulties group was made up of multiple DSM‐5 diagnostic categories, we examined whether any particular category was driving the results. Analyses were repeated for each neurodevelopmental problem category (ID, communication disorders, ASD, ADHD, specific learning disorders, motor disorders).

### Supplementary analyses

Three sensitivity analyses were undertaken. First, to assess the impact of missing data on the definition of our study groups, regression and path analyses were repeated on (a) a sample with complete neurodevelopmental data on all seven indicators (*n* = 3,824) and (b) a sample with neurodevelopmental data on at least one indicator (*n* = 9,977) (see Appendices S1 and S2).

Second, anxiety disorder at the age of 7 was added as a covariate, to establish that any association observed between neurodevelopmental difficulties and depression was explained by irritability rather than co‐occurring anxiety (see Appendix S3 and Table S1).

Third, analyses were repeated using a binary measure of irritability (individuals with no irritability (score = 0) vs. those with any irritability (score ≥ 1)) (see Appendix S4 and Table S2).

### Sample selection

The case‐comparison samples for this study included participants who had any neurodevelopmental difficulty at the age of 7–9 years (*n* = 1,697), and a selected comparison group who had complete data on all the neurodevelopmental measures and thus we could be confident that they had no neurodevelopmental difficulties (*n* = 3,177) (see Figure S1), providing a total sample of 4,874. Of these individuals, 4,512 (92.6%) had irritability data at the age of 7, 2,668 (55%) had outcome data (MDD age 10–15) and 2,546 (52%) individuals had data on all variables included in the model. To minimize the bias from missing data as well as to improve precision, multiple imputation by chained equations was used to impute the missing outcomes (White, Royston, & Wood, 2011).

The imputation model included all variables in the analysis model, variables that predicted missingness in the outcome and variables associated with the outcome (full list of variables available on request). Where continuous variables were not normally distributed, predictive mean matching was applied. Fifty imputed data sets were created using 10 cycles of regression switching. Analyses were run on imputed data sets by combining estimates using Rubin's rules (White et al., 2011).

## Results

### Sample description

Children with neurodevelopmental difficulties at the age of 7–9 were more likely to be male, come from lower social class families, have higher rates of psychopathology, have a higher mean irritability score at the age of 7 and were more likely to be classified as having special education needs by their school than those in the comparison group (Table 2).

**Table 2:** Childhood characteristics of those with neurodevelopmental (ND) difficulties compared to those without

	ND difficulties (*n* ≤ 1,697)	No ND difficulties (*n* ≤ 3,177)	Test statistic
Gender^a^ (% male (*n*))	63.1% (1,071)	47.3% (1,503)	χ^2^ = 110.8, *p* < .001
Social class based on mother's occupation^b^ (% I/II (*n*))	29.2% (407)	40.2% (1,153)	χ^2^ = 49.4, *p* < .001
Oppositional defiant disorder^c^ (% (*n*))	16.4% (228)	0.6% (20)	χ^2^ = 468.8, *p* < .001
Any anxiety disorder^d^ (% (*n*))	4.8% (68)	1.1% (36)	χ^2^ = 60.3, *p* < .001
Major depressive disorder^e^ (% (*n*))	2.2% (30)	0.4% (11)	χ^2^ = 35.7, *p* < .001
Irritable score^f^ (mean)	1.25	0.33	*t*(4,510) = −24.5, *p* < .001
Special educational needs^g^ (% (*n*))	29.9% (403)	3.2% (101)	χ^2^ = 672.5, *p* < .001

Numbers for analysis: ^a^ *n* = 4,874, ^b^ *n* = 4,261, ^c^ *n* = 4,566, ^d^ *n* = 4,582, ^e^ *n *= 4,506, ^f^ *n* = 4,512, ^g^ *n* = 4,488.

Social class based on occupation split into I/II or II/IV/V. DSM‐IV diagnoses of oppositional defiant disorder, any anxiety disorder and major depressive disorder derived using parent‐rated Development and Well‐Being Assessment (DAWBA) at the age of 7. Irritable score calculated using 3 items from parent‐rated DAWBA at the age of 7 (temper outbursts, touchy/easily annoyed, angry/resentful)—possible score = 0–6.

### Childhood neurodevelopmental difficulties and adolescent depression

The neurodevelopmental group had higher rates of depression at the age of 10‐15 than the comparison group (4.5% vs. 2.0%; χ^2^ = 11.48, *p* = .001).

The association between child neurodevelopmental difficulties and later MDD remained significant after removing those with MDD at the age of 7 and controlling for gender, both using complete cases (OR = 2.11, 95% CI = 1.24, 3.60, *p* = .006) and imputed data sets (OR = 2.25, 95% CI = 1.54, 3.29, *p* < .001).

### Contribution of childhood irritability to the association between neurodevelopmental difficulties and depression

There was a significant association between irritability score and the presence of neurodevelopmental difficulties (*r* = .50, *p* < .001).

Irritability at the age of 7 was associated with MDD at the age of 10–15 after controlling for gender and removing those with age 7 MDD, both using complete cases (OR = 1.48, 95% CI = 1.29, 1.72, *p* < .001) and imputed data sets (OR = 1.41, 95% CI = 1.28,1.56, *p* < .001).

**Figure 2:**
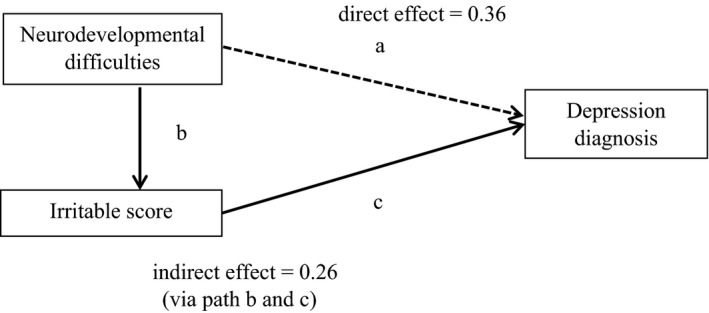
Indirect effect of irritability on the association between neurodevelopmental difficulties and depression. Interrupted line shows a path for which the magnitude of the path coefficient does not reach conventional levels of statistical significance

The hypothesized path diagram included direct and indirect (via irritability) paths from neurodevelopmental difficulties to depression (Figure 2). Using complete cases for analysis, adding irritability reduced the coefficient (log odds) between neurodevelopmental difficulties and depression from .62 (95% CI = 0.06, 1.17, *p* = .029) to .36 (95% CI = 0.23, 0.95, *p* = .234), leaving an indirect effect of .26 (95% CI = 0.14, 0.38, *p* < .001). Overall, 42% of the total effect was explained by irritability.

### Testing individual neurodevelopmental disorders

Analyses of each of the neurodevelopmental categories and depression separately suggested that difficulties in pragmatic language (CCC) and social communication (SCDC) and ADHD (DAWBA symptoms) were associated with later depression (Table 3). Other neurodevelopmental indicators were not associated with later depression.

**Table 3:** Examining association between each neurodevelopmental category and depression age 10–15

	Depression age of 10–15	
	OR (95% CI)	*p* Value
WISC‐III—full‐scale IQ^a^	0.85 (0.20, 3.5)	.824
CCC—speech and syntax subscale^b^	0.98 (0.35, 2.72)	.967
CCC—pragmatic composite subscale^c^	5.12 (2.64, 9.92)	<.001
SCDC^d^	4.63 (2.34, 9.16)	<.001
DAWBA ADHD symptoms^e^	4.27 (2.10, 8.69)	<.001
WORD—basic reading subtest^f^	1.83 (0.65, 5.16)	.256
MABC^g^	1.25 (0.45, 3.50)	.671

ADHD, attention‐deficit/hyperactivity disorder; CCC, Children's Communication Checklist; DAWBA, Development and Well‐Being Assessment; MABC, Movement Assessment Battery for Children; SCDC, Social Communication Disorders Checklist; WISC‐III, Wechsler Intelligence Scale for Children; WORD, Wechsler Objective Reading Dimension. The association between the bottom 5th percentile on each measure of neurodevelopmental difficulties and later depression was examined. Results are for complete cases. Analyses controlled for gender. Those with baseline depression diagnosis were removed from analysis.

^a^ *n* = 2,604, ^b^ *n* = 2,634, ^c^ *n* = 2,581, ^d^ *n* = 2,600, ^e^ *n* = 2,616, ^f^ *n* = 2,599, ^g^ *n* = 2,538.

For the measures of ASD and ADHD, irritability explained a large proportion of the association. Irritability explained 29% of the association between pragmatic language difficulties and depression (indirect effect: log odds = 0.39, 95% CI = 0.20, 0.59, *p* < .001), 51% of the association between social communication difficulties and depression (indirect effect: log odds = 0.73, 95% CI = 0.31,1.14, *p* = .001) and 42% of the association between ADHD problems and depression (indirect effect: log odds = 0.55, 95% CI = 0.27, 0.83, *p* < .001).

### Supplementary analyses

Alternative ways of defining the study groups using either complete or any available neurodevelopmental data, respectively, yielded very similar findings to the study full sample (see Appendices S1 and S2).

Controlling for anxiety disorder did not affect the results (see Appendix S3 and Table S1). Finally, the pattern of results using a dichotomous measure of (any vs. no) irritability was the same as that observed for the continuous measure (see Appendix S4 and Table S2).

## Discussion

Using a longitudinal design, we observed that neurodevelopmental difficulties in childhood are associated with later adolescent depression and that a significant proportion of this association is explained by childhood irritability. In fact, when we take into account irritability, operationalized using the irritable dimension of ODD, the magnitude of association between neurodevelopmental difficulties and depression drops below the threshold for statistical significance. This suggests that irritability is a major contributor in explaining the link between neurodevelopmental problems and later depression.

Previous studies have found that individuals with neurodevelopmental disorders are at elevated risk of depression. Most of this research examined the associations between ADHD or ASD and depression (Daviss, 2008; Ghaziuddin, Ghaziuddin, & Greden, 2002; Kim et al., 2000; Meinzer et al., 2016). However, associations between other neurodevelopmental disorders and depression have also been found (e.g. reading and tic disorders) (Gadow et al., 2012; Mammarella et al., 2016). Our results are partially consistent with this literature, suggesting that while children with neurodevelopmental difficulties are at increased risk of later depression, findings may vary for specific problem types. This is clinically relevant, in terms of understanding which children with neurodevelopmental difficulties are at high risk of depression; regardless of the primary presenting problem; and elevated levels of ASD/social communication and ADHD symptoms appear to be associated with greatest risk.

Our second aim involved examining the contribution of irritability to the association between neurodevelopmental difficulties and depression. The results suggest that irritability, when measured as an irritable dimension of ODD, plays an important role. Previous studies have shown that irritability is an important predictor of future depression in the general population (Althoff, Kuny‐Slock, Verhulst, Hudziak, & van der Ende, 2014; Stringaris et al., 2009). Our results suggest it is also important for those with neurodevelopmental difficulties. Even when controlling for anxiety, the contribution of irritability was important, with 42% of the association between neurodevelopmental difficulties and depression explained by irritability.

These findings are clinically relevant. Identifying irritability in children with neurodevelopmental difficulties (particularly those with ADHD and ASD) may help to identify those at risk of later depression. This may help with early identification and treatment of depression in a group where depression is common and impairing (Daviss, 2008; Ghaziuddin et al., 2002). It may also provide an opportunity to prevent the onset of depression, for example by treating irritability early.

Understanding the mechanisms underlying irritability and its association with depression will inform the development of effective treatments. Literature suggests that genetic factors may be important. Family history of depression has been associated with irritability in a general population sample (Krieger, Leibenluft, et al., 2013) and a clinical ADHD sample (Eyre et al., 2017). Twin studies suggest irritability and depression have common genetic underpinnings (Savage et al., 2015; Stringaris, Zavos, Leibenluft, Maughan, & Eley, 2012). Environmental factors may also be of relevance in the association between irritability and depression. For example, studies of adolescent depression have found stressful life events, particularly stressors affecting relationships, to be important risk factors (Thapar et al., 2012). Irritability is associated with significant impairment in multiple areas of functioning including family relationships (Copeland, Angold, Costello, & Egger, 2013). Therefore, irritability (including the irritable dimension of ODD) could predispose to family relationship problems, which may predispose to depression. If this was the case, interventions such as parent training may be of benefit. Indeed, the effectiveness of parenting interventions has been well established in ODD (Scott & Gardner, 2015). However, more research is needed to test whether such interventions prevent depression onset in those with neurodevelopmental difficulties. Further research is also needed to examine whether mechanisms underlying irritability and their possible links with depression differ in young people with neurodevelopmental difficulties compared to those without.

### Study limitations

Although the use of a large longitudinally assessed sample was a strength of the study, several limitations should be mentioned. As for any large longitudinal cohort study, there was a significant proportion of missing data in the outcome (MDD age 10–15). However, the pattern of results remained the same for complete case analyses and imputed data. Also, the way in which the neurodevelopmental difficulties categorical variable was derived meant that, in order to be categorized as having any neurodevelopmental difficulty, data on a minimum of one measure of neurodevelopmental difficulties were needed. However, to be categorized as having no neurodevelopmental difficulty, data on all neurodevelopmental measures were necessary. Despite this, the pattern of results remained the same when sensitivity analyses were undertaken firstly, including only those with complete data and secondly, allowing missing data in both neurodevelopmental and comparison groups.

There are also limitations in the measures used. The neurodevelopmental grouping variable aimed to cover a broad range of difficulties based on the DSM‐5 neurodevelopmental disorder categories. Symptoms in most but not all of the categories listed in DSM‐5 were included due to the available measures in the ALSPAC sample. Also, for the outcome, even though the measurement of depression across 3 time points was a strength of the study, the rater changed from parent report to self‐report at the age of 15. However, this reflects clinical practice, where there is greater reliance on parental reports in younger children and self‐reports in adolescents. It should also be noted that, although removing children with MDD at the age of 7 ensured that depression diagnosis did not precede the assessment of neurodevelopmental difficulties and irritability, children may still have experienced depression symptoms prior to this without meeting diagnostic criteria. Finally, our findings cannot automatically be generalized to clinical samples; further longitudinal research in clinical samples is needed.

## Conclusion

This longitudinal study suggests that children with neurodevelopmental difficulties, specifically autistic and ADHD problems, are at increased risk of developing later adolescent depression. We found that irritability was an important contributor to this association. This suggests that the high rates of irritability known to be present in those with neurodevelopmental problems might explain the high rates of depression in this group. The next step is to identify the mechanisms involved in this association, which could facilitate the search for effective interventions.

**Key points**

Childhood neurodevelopmental difficulties, particularly ASD/social communication and ADHD problems, are associated with later adolescent depression.
Irritability explains a significant proportion of the association between neurodevelopmental difficulties and adolescent depression.
As irritability precedes the onset of depression, there is opportunity for early identification, treatment and prevention of depression.
Future research is needed to understand mechanisms involved, to develop effective interventions and test existing ones.

## Supporting information


**Appendix S1.** Analysis for those with complete neurodevelopmental data.
**Appendix S2.** Analysis for those with missing neurodevelopmental data.
**Appendix S3.** Controlling for anxiety.
**Appendix S4.** Analyses using binary measure of irritability.
**Table S1.** Path analysis examining the association between ND difficulties and depression, after controlling for anxiety disorder (in addition to gender and removing those with baseline depression diagnosis) at age 7.
**Table S2.** Path analysis examining the association between ND difficulties and depression, using a binary measure of irritability.
**Figure S1.** Flow chart showing how the sample for analysis was selected.
