# Supplementary material for: Childhood neurodevelopmental difficulties and risk of adolescent depression: the role of irritability
Source: J Child Psychol Psychiatry. 2019 Mar 25;60(8):866–74. doi: 10.1111/jcpp.13053 (PMC6767365; doi:10.1111/jcpp.13053)
Supplement: Supplementary file 1 — Appendix S1. Analysis for those with complete neurodevelopmental data. Appendix S2. Analysis for those with missing neurodevelopmental data. Appendix S3. Controlling for anxiety. Appendix S4. Analyses using binary measure of irritability. Table S1. Path analysis examining the association between ND difficulties and depression, after controlling for anxiety disorder (in addition to gender and removing those with baseline depression diagnosis) at age 7. Table S2. Path analysis examining the association between ND difficulties and depression, using a binary measure of irritability. Figure S1. Flow chart showing how the sample for analysis was selected. [file JCPP-60-866-s001.docx]

**Supporting information - Childhood neurodevelopmental difficulties and risk of adolescent depression: the role of irritability – by Eyre, et al.**

**Figure S1.** Flow chart showing how the sample for analysis was selected.

Identifying sample for analysis:

ND difficulties data available for:

1 measure **n= 296**

2 measures **n= 954**

3 measures **n= 536**

4 measures **n= 1061**

5 measures **n= 951**

6 measures **n= 1305**

No ND difficulties present on 1-6 ND measures at age 7-9 **n= 5103**

ALSPAC sample

**n= 13,975**

ALSPAC sample with data on at least one ND measure at age 7-9 **n= 9977**

**Selected comparison group:**

No ND difficulties present using all 7 ND measures at age 7-9 **n= 3177**

**ND difficulties group:** ND difficulties present on >=1 ND measure at age 7-9

**n= 1697**

**Main sample used for analysis n= 4874**

**Appendix S1.** Analysis for those with complete neurodevelopmental data.

When including those with complete data on neurodevelopmental measures to make the binary neurodevelopmental difficulties variable, the sample size was reduced to 3824 (number with neurodevelopmental difficulties=647, number without neurodevelopmental difficulties=3177 (i.e.17% of the sample with complete data had neurodevelopmental difficulties)).

In this sample, childhood neurodevelopmental (ND) difficulties continued to be associated with depression diagnosis age 10-15, when controlling for gender and after removing those with baseline depression diagnosis: OR=2.61, 95% CI=1.46, 4.69; p=0.001. The results from the path model found that adding irritability reduced the coefficient between ND difficulties and depression from 0.86 (95% CI=0.26, 1.47; p=0.005) to 0.61 (95% CI=-0.04, 1.26; p=0.064), leaving an indirect effect of 0.25 (95% CI=0.11, 0.39; p<0.001). A total of 29% of the total effect was explained by irritability.

**Appendix S2.** Analysis for those with missing neurodevelopmental data.

When including those with data on at least one measure of neurodevelopmental difficulties for either the neurodevelopmental difficulties group or the comparison group, the sample size was increased to 9977 (number with neurodevelopmental difficulties=1697, number without neurodevelopmental difficulties=8280).

Using this sample, childhood neurodevelopmental (ND) difficulties continued to be associated with depression diagnosis age 10-15 when controlling for gender and after removing those with baseline depression diagnosis: OR=2.32, 95% CI=1.47, 3.66; p<0.001. The results from the path model found that adding irritability reduced the coefficient between ND difficulties and depression from 0.65 (95% CI=0.14, 1.16; p=0.013) to 0.43 (95% CI=-0.11, 0.97; p=0.120), leaving an indirect effect of 0.22 (95% CI=0.11, 0.33; p<0.001). A total of 34% of the total effect was explained by irritability.

**Appendix S3.** Controlling for anxiety.

Childhood ND difficulties continued to be associated with depression diagnosis age 10-15, after controlling for baseline anxiety disorder (in addition to gender and removing those with baseline depression diagnosis). Analyses using complete cases: OR=1.97, 95% CI=1.15, 3.38; p=0.013; analyses using imputed datasets: OR=2.15, 95% CI=1.46, 3.15; p<0.001.

Using the path model in complete cases, irritability explained 42% of the association between ND difficulties after controlling for baseline anxiety disorder. Supplementary table S1 shows the association between ND difficulties and depression decomposed into total, direct and indirect effect (through irritability).

**Table S1****.** Path analysis examining the association between ND difficulties and depression, after controlling for anxiety disorder (in addition to gender and removing those with baseline depression diagnosis) at age 7.

| **Path** | **Coefficient**  **(log odds)** | **95% CI** | **P value** |
| --- | --- | --- | --- |
| **Total effect** | 0.58 | 0.015, 1.14 | 0.044 |
| **Direct effect** | 0.33 | -0,26, 0.93 | 0.270 |
| **Indirect effect** | 0.24 | 0 0.08, 0.40 | 0.004 |

**Appendix S4.** Analyses using binary measure of irritability.

When a binary measure of irritability was used in the path analysis, this measure explained 40% of the association between neurodevelopmental difficulties and later depression (controlling for gender and after removing those with baseline depression diagnosis). Supplementary table S2 shows the association between ND difficulties and depression decomposed into total, direct and indirect effect (through a binary measure of irritability).

**Table S2.** Path analysis examining the association between ND difficulties and depression, using a binary measure of irritability.

| **Path** | **Coefficient**  **(log odds)** | **95% CI** | **P value** |
| --- | --- | --- | --- |
| **Total effect** | 0.71 | 0.17, 1.25 | 0.009 |
| **Direct effect** | 0.43 | -0.13, 0.99 | 0.131 |
| **Indirect effect** | 0.28 | 0.14, 0.43 | <0.001 |
